# Transformer-based progressive residual network for single image dehazing

**DOI:** 10.3389/fnbot.2022.1084543

**Published:** 2022-12-06

**Authors:** Zhe Yang, Xiaoling Li, Jinjiang Li

**Affiliations:** ^1^School of Computer Science and Technology, Intgrow Education Technology, Qingdao Vocational and Technical College of Hotel Management, Shandong Technology and Business University, Yantai, China; ^2^Institute of Artificial Intelligence, University of Science and Technology Beijing, Beijing, China; ^3^Co-Innovation Center of Shandong Colleges and Universities, Future Intelligent Computing, Shandong Technology and Business University, Yantai, China

**Keywords:** transformer, residual network, image dehazing, progressive recurrent, multiple self-attention

## Abstract

**Introduction:**

The seriously degraded fogging image affects the further visual tasks. How to obtain a fog-free image is not only challenging, but also important in computer vision. Recently, the vision transformer (ViT) architecture has achieved very efficient performance in several vision areas.

**Methods:**

In this paper, we propose a new transformer-based progressive residual network. Different from the existing single-stage ViT architecture, we recursively call the progressive residual network with the introduction of swin transformer. Specifically, our progressive residual network consists of three main components: the recurrent block, the transformer codecs and the supervise fusion module. First, the recursive block learns the features of the input image, while connecting the original image features of the original iteration. Then, the encoder introduces the swin transformer block to encode the feature representation of the decomposed block, and continuously reduces the feature mapping resolution to extract remote context features. The decoder recursively selects and fuses image features by combining attention mechanism and dense residual blocks. In addition, we add a channel attention mechanism between codecs to focus on the importance of different features.

**Results and discussion:**

The experimental results show that the performance of this method outperforms state-of-the-art handcrafted and learning-based methods.

## 1. Introduction

Due to the color distortion, blurring and other quality problems of haze images that affect further information capture, single image deblurring has always been a challenging and highly concerned problem. The deblurring method originates from the classical atmospheric scattering model, and the imaging formula is as follows:


(1) $$\begin{array}{l} \begin{array}{c} I(x)=J(x)t(x)+A(1-t(x)),\\ \;t(x)=e^{-\beta (\lambda )d(x)},\; \end{array} \end{array}$$

where *I*(*x*) is the degraded image, *J*(*x*) is the brightness of the scene when it does not propagate through the water, *t*(*x*) is the transmissivity of the propagation medium, β(λ) is the attenuation coefficient of different wavelengths of light, λ represents different color channels, *d*(*x*) is the distance between the camera and objects, and *A* is the ambient atmospheric light of the scene. Many deblurring methods based on imaging models (He et al., 2010; Zhu et al., 2015; Berman et al., 2016, 2018; Middleton, 2019) restore clean images by reversing the blurring process, in which the atmospheric channel A (x) and the medium transmission map t(x) need to be estimated by manual prior. Although the quality of the blurred image is improved to some extent, these physical priors are not always reliable, and without priors and constraints, the blurring performance will be further reduced, resulting in artifacts and color distortion.

With the development of deep learning in recent years, convolutional neural network has become the backbone of various visual tasks due to its robustness and accuracy. The progress of CNN architecture improves network performance and promotes the progress of single image defogging (Qin et al., 2020) and other hierarchical visual tasks (Afshar et al., 2020; El Helou and Süsstrunk, 2020; Akbari et al., 2021). Although the method based on CNN has special representational ability. It is unable to learn global and remote semantic information interaction well due to the localization of convolution operation. To overcome these problems, some methods add self-attention mechanism (Wang et al., 2020). While others use full attention structure to replace traditional RNN modeling, and propose transformer model to solve Seq2Seq problem (Vaswani et al., 2017). Compared to CNN, Transformer does not increase to distance from the number of operations required to calculate the association between two positions, and can not only do parallel calculations, but also efficiently process global information and encode longer sequences. Due to its powerful presentation capabilities, researchers have applied Transformer to computer vision tasks such as image representation (Wu et al., 2020), image segmentation (Zheng et al., 2021), object detection (Carion et al., 2020; Zhu et al., 2020), pose estimation (Huang et al., 2020a,b; Lin et al., 2021b) and pre-training (Chen et al., 2021a). There are still some problems that can not be ignored when the model is transferred to the visual task, such as the large scale change of the visual target and the high resolution pixel of CV.

Recently, researchers have improved Vit, and swin transformer (Liu et al., 2021) has solved these problems and proved its effectiveness and superiority in target detection, instance segmentation, semantic segmentation and other task fields. Therefore, some methods uses it as the backbone for image classification, image restoration and medical image segmentation. For example, Chen et al. (2021b) introduces a transformer to encode image features and extract contextual input sequences. Cao et al. (2021) proposes a pure transformer similar to u-net for medical image segmentation. Input tokenized image patches to a transformer-based u-shaped encoder-decoder architecture with skip-connections for local-global semantic feature learning. Liang et al. (2021) uses several swin Transformer layers and a residual swin transformer block with a residual connection for image restoration. In order to obtain image features from multi-scale, Gao et al. (2021) proposes a method combining swin transformer trunk and traditional multi-stage network, which effectively improved the ability of feature extraction. Yue et al. (2021) proposes an iterative and progressive sampling strategy and combined with the transformer to classify images.

Inspired by the above process, we proposed an progressive residual network (PRnet) based on swin transformer. PRnet consists of recurrent block, transformer codecs and supervised fusion modules. First, we have a recurrent block that learns shallow features of input images and introduces a long short-term memory (LSTM) network to connect different iterations, ensuring that more of the original image features can be retained over multiple iterations of the model. The transformer codec then learns the sequence representation of the input image through the u-net structure, and effectively extracts the remote context features from multiple scales of the image. The encoder introduces swin transformer block to encode feature representation from the decomposed patch, and continuously reduces the resolution of feature map for local relationship modeling. Decoder decodes hidden features through convolution and upsampling and realizes dimensional transformation to further predict the semantic output of the global context representation. In addition, we connect the encoders through skip connection and add channel attention. this design can effectively avoid the loss of original features and improve the quality of the output image. Finally, the supervised fusion module combines the attention mechanism and dense residual blocks to recursively select and fuse the image features and transfer the attention-guided features to the next stage, which can effectively preserve the original features of the image and prevent the model from over-fitting. In addition, the whole recursive process under the supervision of the original input image can effectively retain the original resolution characteristics of the image, improve the learning efficiency and defogging performance of the network.

**Figure 1:**
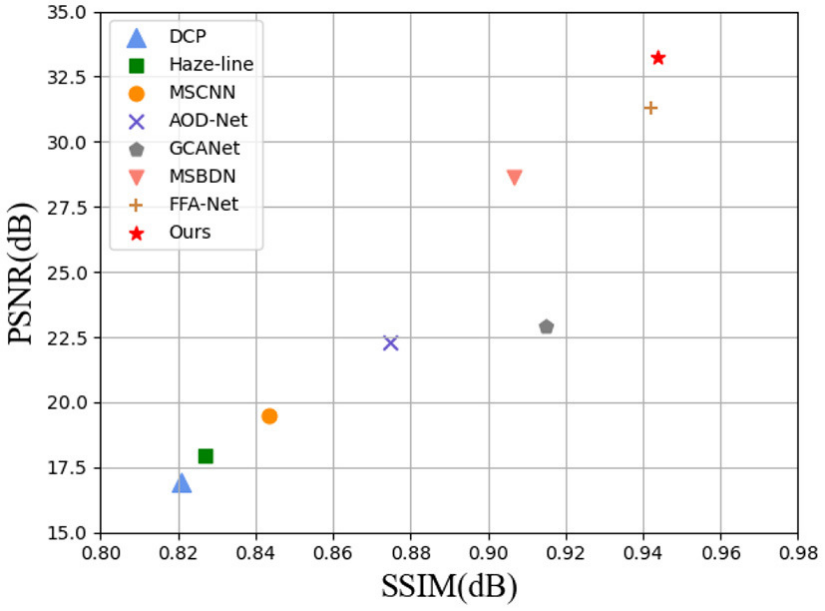
Image dehazing on the RESIDE dataset (Li et al., 2018). Under different evaluation indexes, the performance of our method is the most advanced (SSIM on x-axis and PSNR on y-axis) when compared with several advanced methods.

To validate our approach, we tested it on different data sets. A large number of experiments and qualitative and quantitative evaluations show that our iterative strategy is beneficial to image restoration and is superior to other state-of-the art methods (see Figure 1). In short, our contribution is:

- We introduce the swin transformer into the iterative progressive residual network (PRnet), which obtains sufficient contextual semantic information and spatial features by learning multi-scale feature information of the input image.
- We introduce channel attention between the encoder and decoder, which makes the module focus on extracting significant useful features related to clean image in the input image.
- We design a supervised fusion module, which combined the dense residual block with attention to conduct recursive supervised fusion of image features under the supervision of ground-truth.

## 2. Related work

In this section, we will conduct a comprehensive review of fog removal methods and vision transformer relevant to our work. We will conduct a comprehensive review of single image defogging algorithms, including traditional image defogging and deep learning-based image defogging methods.

### 2.1. Model-based method

By observing and analyzing the imaging process of fog image and its relationship with clean image, the physical model of atmospheric scattering for fog imaging is established. The model-based method tries to estimate the atmospheric light and medium transmission map using the handmade prior knowledge, and then restore the blurred image. Dark channel prior (DCP) is one of the outstanding representatives of priority-based methods. He et al. (2010) assumed that each pixel with a value close to zero has at least one color channel, and combined it with haze imaging model to recover high-quality fog-free images. Zhu et al. (2015) proposed a method of restoring image color attenuation by establishing a linear model to estimate the depth of field information. Berman et al. (2016, 2018) propose an algorithm based on non-local prior to predicting atmospheric light by identifying haze lines and estimating transmission per pixel. Although these methods have achieved some success, they are still constrained by prior knowledge, which may lead to insufficient demisting effect and more serious artifacts and blurriness.

### 2.2. Deep-learning method

In recent years, a large number of methods based on deep learning have flooded with the field of dehazing. Some deep learning methods still combine physical models or prior knowledge to improve the accuracy of fog removal. Kar et al. (2020) takes the atmospheric light and transmission diagrams estimated by convolutional architecture as a prior condition, and uses an iterative mechanism to gradually update the estimated value to the more appropriate estimated value of fuzzy conditions. Yan et al. (2020) uses multi-scale convolutional neural network combined with atmospheric scattering model to extract features of different scales from global to local. By learning the mapping relationship between hazy images and their transmission images, Ren et al. (2020) predicts projected images at multiple scales and refined the results of defogging. Different from the above methods, Anvari and Athitsos (2020), Liu et al. (2020b), Wang et al. (2021), and Zhang et al. (2022) directly restores blurred images end-to-end by learning the mapping between blurred and clear images. Anvari and Athitsos (2020) combines encoder-decoder structure and residual block to restore fog-free scenes. Through local residual learning and feature attention mechanism, Qin et al. (2020) designs an end-to-end feature fusion attention network to directly restore fog-free images. Liu et al. (2020b) uses residual blocks in fine-grained and coarse-grained networks to generate clean images directly from input fuzzy images. These methods use residual learning to enable network residual links to bypass unimportant information and enable the network architecture to focus on more effective information.

In addition, some methods take into account the morphological differences of fuzzy images at different scales to extract, transfer and fuse multi-scale image features. For example, Yeh et al. (2019) relies on multi-scale residual learning and image decomposition to remove haze from a single image, and feature transmission benefited from the basic components of remnant CNN architecture and simplified u-net structure. Liu et al. (2019) performs multi-scale estimation based on attention, alleviates the bottleneck problem of traditional multi-scale methods and reduces the output image artifacts. Li et al. (2021) designed a dual attention to extract global features and guide subsequent recursive units. Through the strengthen-operate-subtract boosting strategy, Dong et al. (2020) proposes a multi-scale enhanced defogging network with dense feature fusion based on u-net architecture. Despite its success, the limitations of the convolution layer, the main building block of CNN networks, limit the ability to learn remote spatial relevance in such networks. To solve these problems, we have introduced the swin transformer block in this paper.

### 2.3. Vision transformer

Transformer was first proposed for machine translation Vaswani et al. (2017) and is widely used in many natural languages processing tasks. Because of its powerful representation ability, it has recently been applied to computer vision tasks. To adapt transformer for visual tasks, the researchers have modified it. For example, Transformer model does not have translation invariance and locality like CNN. Parmar et al. (2018) applies self-attention to local fields and solves the problem that it cannot be well generalized to new tasks when data is insufficient. In addition, location information is very important for Transformer. Dosovitskiy et al. (2020) adds position embedding to feature vector and proposes a visual transformer (ViT), which directly applies pure transformer to image patch sequence to complete image classification task. In addition, Transformer model does not have translation invariance and locality like CNN. So it cannot be generalized to new tasks when data is insufficient. Liu et al. (2021) improves ViT by limiting self-attention computation to non-overlapping local windows and allowing cross-window connections to improve efficiency. This layered architecture has the flexibility to model at a variety of scales, which can be well generalized to new tasks. For example, with Swin Transformer as its backbone, Xie et al. (2021) uses self-supervised learning methods to handle object detection and semantic segmentation tasks. Cao et al. (2021) proposes a pure Transformer similar to u-net for medical image segmentation based on u-encoder-decoder architecture and learning local and global semantic features by skipping connections. Huang et al. (2022) has designed an adaptive group attention for Swin Transformer, which reduces the model parameters while taking into account the network performance. Lin et al. (2021a) tries to incorporate the advantages of layered Swin Transformer into the standard encoder and decoder U-shaped architecture at the same time, so as to improve the semantic segmentation quality of different medical images. It designes a strong baseline model for image recovery based on Swin Transformer, and combined Swin Transformer layer with residual connection for depth feature extraction. The success of Swin Transformer in these visual tasks proves that it is superior in some respects to the full convolution approach.

## 3. Progressive image dehazing networks

**Figure 2:**
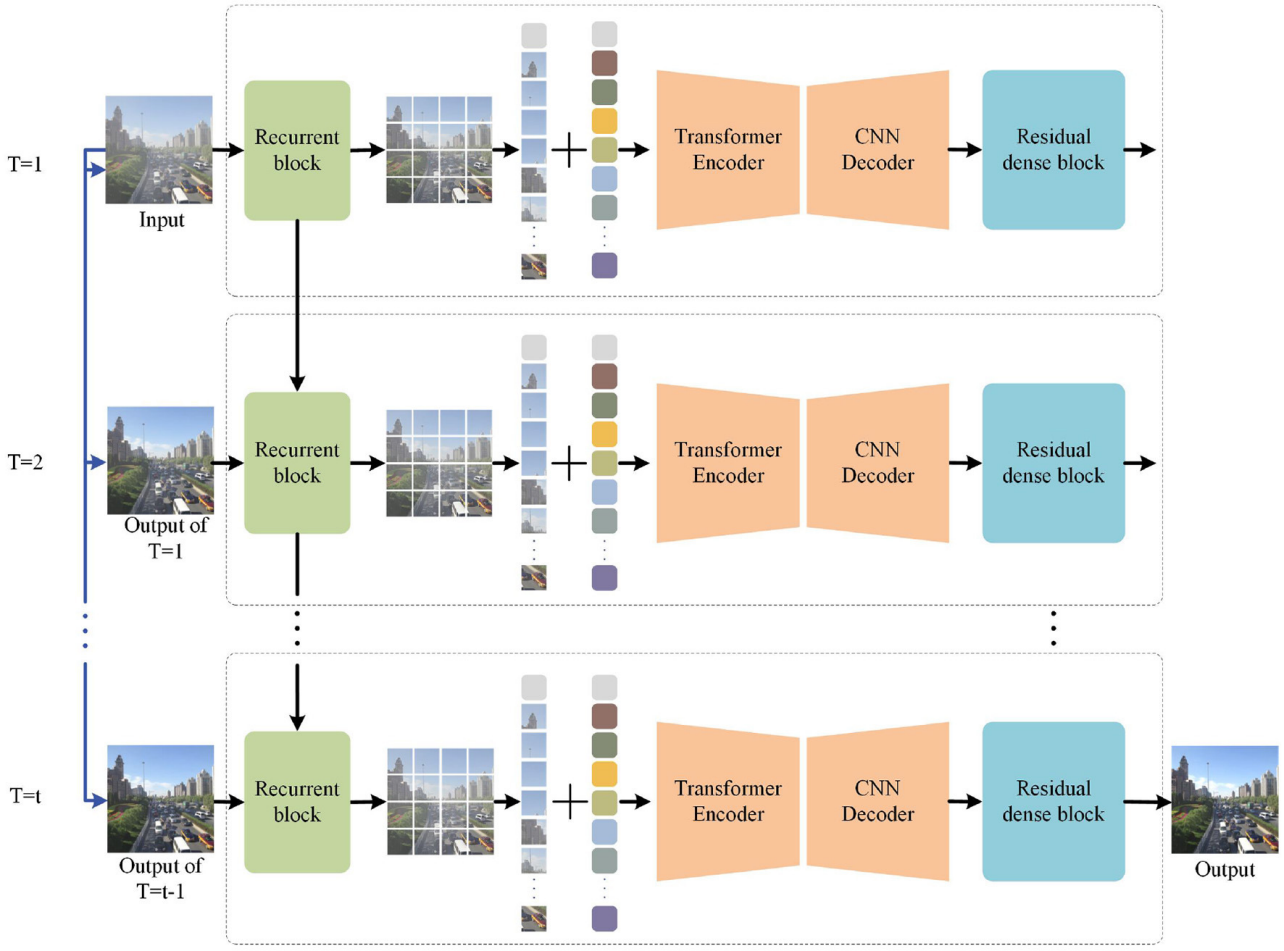
The proposed framework for PRnet. PRnet extracts early features through recurrent blocks, then extracts multi-scale features through transformer codec, and finally integrates the features into the supervised fusion module. The blue line represents concatenate operation, and the black line represents forward.

In this section, we first introduce the cross scales supervisory integration mechanism (CSSI) and then introduce our overall architecture of progressive residual networks. As shown in Figure 2, it is made up of recurrent block, a Transformer encoder-decoder module based on the u-net architecture, and a supervised fusion module. Finally, we will describe the details of each module and the loss function in detail.

### 3.1. Cross scales supervisory integration mechanism

**Figure 3:**
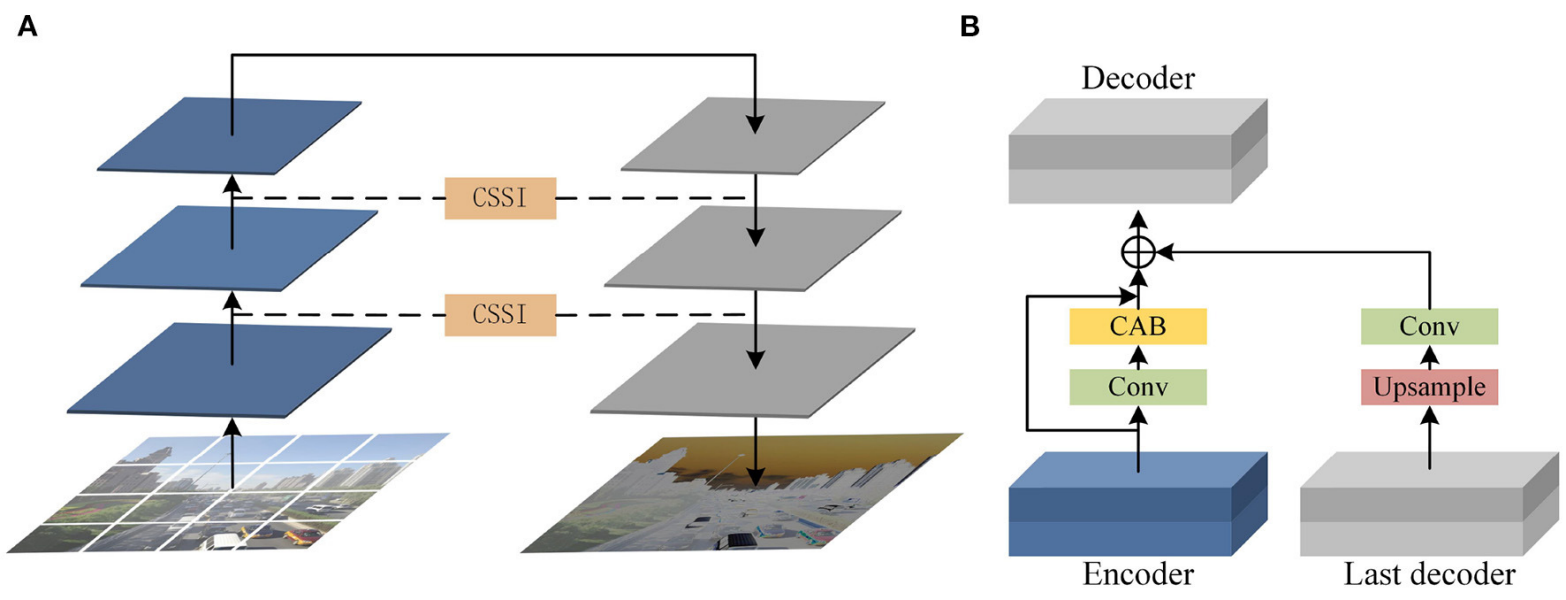
**(A)** Encoder-decoder block. **(B)** Cross scale supervisory integration mechanism between encoder decoder and the last decoder.

Our analysis shows that if the encoder and decoder are independent from each other, multi-scale features cannot interact with each other, which will greatly reduce the performance of the model (Figure 3). If features are fused through simple transfer, convolution or addition microstructures, and these features are treated equally, it is easy to cause redundancy and bring great burden to the network. To solve this problem, we added cross scales supervisory integration (CSSI) between encoders, which can improve the learning efficiency of U-codecs, make full use of features of different scales, and ensure the connectivity of the model. CSSI converts the output feature of encoder layer through 1 × 1 convolution. Then, the convolution features are paid attention to the information useful to the current output features through the channel attention block (CAB). The channel attention mechanism aggregates spatial dimension features using operations such as convolution, activation function, global average pooling and maximum pooling. Subsequently, the above features are fused through the following skip connection:


(2) $$\begin{array}{l} \begin{array}{c} F_{i}=C_{i}⊕E_{i}=CAB\left[\text{conv}\left(E_{i}\right)\right]⊕E_{i}, \end{array} \end{array}$$

where *E*_*i*_ and *C*_*i*_ represent the output of the encoder layer and channel attention mechanism respectively. Next, the output feature of the encoder layer is fused with the up-sampling and convolution operation results of the previous decoder layer to obtain the input feature of the next decoder layer:


(3) $$\begin{array}{l} \begin{array}{c} D_{i}=CSSI\left[F_{i}\text{,conv}\left(↑D_{i-1}\right)\right], \end{array} \end{array}$$

where *D*_*i*−1_ and *D*_*i*_ represent the features of the previous and next decoder layers.

CSSI explores the relationship between feature maps of different channels through channel attention, adjusts and aggregates different feature maps in the process of feature interaction, and finally transfers them to the decoder layer. On the one hand, CSSI makes the network pay more attention to find the significant useful information related to the current output in the input data, which can effectively avoid the loss of original features and improve the quality of the output image. On the other hand, CSSI can improve the efficiency of feature fusion and interaction between codecs with different resolutions, effectively reducing the network burden.

### 3.2. Progressive networks

Swin Transformer interacts with the global information of the image, without considering the importance of the content of the image area and the overall structure of the object, and cannot pay better attention to the structure and details of the image. In order to make up for the above defects, we propose a new progressive residual network (PRnet), which solves the problem of fog removal through multiple stages. At the same time, u-transformer encoder-decoder is used in each stage to learn the morphological features of foggy images at different scales. To avoid the increase and over-fitting of network parameters, different from the previous multi-stage, we do not pile up several sub-networks, but use the recursive calculation between stages to share the same network parameters in multiple stages. In addition, while swin transformer avoids the segmentation edge loss problem, the Transformer image is smaller than the original image resolution. Therefore, ground truth is used to supervise the network, which can suppress features with less information in the current stage and only allow useful features to be transmitted to the next stage.

#### 3.2.1. Progressive recurrent block

**Figure 4:**
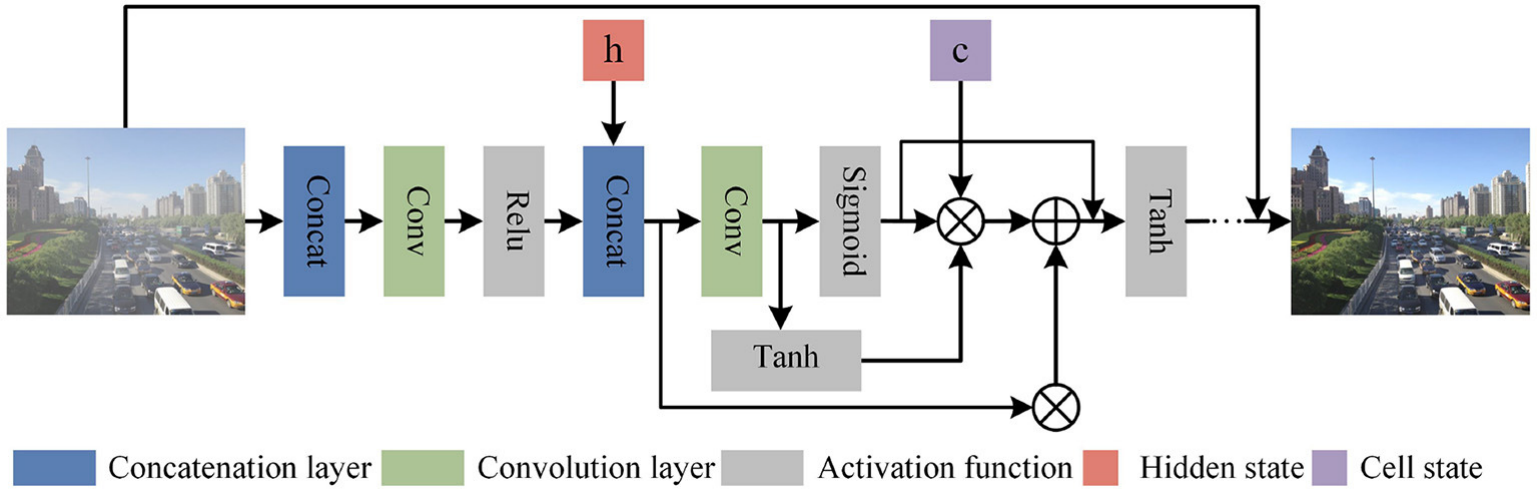
Progressive recurrent block structure. ⊗ Represents Hadamard Product, and the corresponding elements in the matrix are multiplied. ⊕ Represents matrix addition operation.

We designed a Recurrent block in PRnet to learn the shallow features of the input image, and introduced the Long Short-Term Memory (LSTM) (Yamak et al., 2019) networks to connect different iterations to ensure the propagation of features across multiple stages of the model. In the process of feature dependence, more original image features can be retained. As shown in Figure 4, taking the t iteration as an example, we input the original foggy image and the predicted image output by the iteration into the network together, go through the convolutional layer 3 × 3 × 64 with a step size of 1, and then go through the activation function(ReLU) performs nonlinear correction. In the subsequent convolution, we did not perform batch normalization, but added an LSTM layer. LSTM introduces and splices the feature map output *x*_*t*−1_ from the *t*−1 iteration and the previous hidden state *h*_*t*−1_. The feature graph *i*_*t*_ is obtained by convolution, which is used to determine which information is important and needs to be retained. Then feature graphs *f*_*t*_ and *o*_*t*_ controlling forgotten data were obtained through sigmoid activation function, and then forgetting and remembering were carried out according to the following formula:


(4) $$\begin{array}{l} \begin{array}{c} C_{t}=f_{t}*C_{t-1}+i_{t}*\overset{\sim }{\underset{t}{C}}, \end{array} \end{array}$$

Among them, $\overset{\sim }{\underset{t}{C}}$ represents the cell state, which is a feature map obtained by passing *h*_*t*−1_ and *x*_*t*−1_ to the Tanh function. Next, multiply *o*_*t*_ with *C*_*t*_ after Tanh activation to obtain *h*_*t*_ to determine the information carried in the hidden state, namely:


(5) $$\begin{array}{l} \begin{array}{c} h_{t}=o_{t}*\text{Tanh}\left(C_{t}\right), \end{array} \end{array}$$

where *h*_*t*_ is output as the current cell, which is passed to the next time period with the new cell state *C*_*t*_. The output of the entire asymptotic recursive process can be expressed as:


(6) $$\begin{array}{l} \begin{array}{c} f_{res}=LSTM\left(x_{t-1},h_{t-1}\right) \end{array} \end{array}$$

#### 3.2.2. Transformer encoder-decoder

As we all know, multi-scale networks can not only extract low-level high-resolution features and texture detail information, but also extract high-level feature semantic information, and fully extract and utilize image features at different scales. Therefore, we combine the advantages of swin transformer and cnn to design encoder-decoder based on u-net architecture. By learning the sequence representation of the input image, we can ensure that sufficient contextual semantic information and spatial features are acquired during the long-distance transmission.

Swin transformer introduces the locality idea in the Multiple Self-Attention (MSA) module to perform self-attention computation in the window region without overlap. Because of its hierarchical design and generalization, it has proven its effectiveness in several fields such as object detection, semantic segmentation and image denoising. Therefore, we apply Swin transformer directly in encoder to encode the feature representation from the decomposed patch.

Our encoder generates different number tokens through three layers of encoder layer. The first, second and third layers generate $\frac{H}{4}\times \frac{W}{4}$, $\frac{H}{8}\times \frac{W}{8}$, and $\frac{H}{16}\times \frac{W}{16}$ tokens respectively. Each stage consists of Patch Merging and some Swin Transformer Blocks. We merged the image resolution by a sliding window operation for Patch Merging, and divided the image with a given size of *H*×*W* into RGB image patches, and marked them as the original pixel Mosaic vector with a size of 4 × 4. It is then mapped to a vector of dimension 4C using linear embedding. At this time, the output dimension is set to 2C and the feature size is set to $\frac{H}{8}\times \frac{W}{8}$ from the original$\frac{H}{4}\times \frac{W}{4}$. Next, the output feature *z*^*l*−1^ enters two consecutive Swin Transformer Blocks for feature transformation. Unlike MSA in ViT, Swin Transformer Block computes self-attention by adding a relative position bias B to the corresponding head, then the output feature *z*^*l*−1^ of layer *l* can be written as follows:


(7) $$\begin{array}{l} \begin{array}{c} z_{m}^{l}=SW-MSA(LN(z^{l-1}))+z^{l-1},\\ \;z^{l}=MLP(LN(z_{m}^{l}))+z^{l},l=1,2,3, \end{array} \end{array}$$

where $z_{m}^{l}$ represent the output of multi-head self-attention, *z*^*l*^ represent the output of MLP.

Corresponding to the encoder, a symmetric decoder is constructed based on the swin transformer, forming an encoder-decoder based on the u-net architecture.To recover the spatial order, we use a convolution module and upsampling to form a Decoder layer. In the first layer the hidden features are first decoded by bilinear upsampling of the input features ($\frac{H}{16}\times \frac{W}{16}\times 4C$).And then implement dimension transformation in the convolution module. A linear layer is applied to map the dimensions to 2C, then the resolution is extended to $\frac{H}{8}\times \frac{W}{8}$, and finally the output feature ($\frac{H}{8}\times \frac{W}{8}\times 2C$) is fed into the next Decoder layer. Bilinear up-sampling operation can ensure the same dimensions before and after the fusion, so that the fusion and feature mapping under the same dimension can be carried out again. In addition, Decoder decodes hidden features while further predicting the semantic output of the global context representation.

#### 3.2.3. Supervise fusion module

First, the output features of Swin transformer decoder are supervised by ground-truth and attention maps are generated by Supervised Attention (Zamir et al., 2021) to assist the delivery of useful features and effectively preserve the original features of the image. Next, we introduce residual blocks to learn deeper features. Inspired by Kim et al. (2016), we use recursion to unfold the residual block by calling the residual block 5 times, with both input and output channels of 64 and a convolution kernel size of 3 × 3. In addition, a skip connection is used in the residual block to connect the input and output, which is then passed to the next residual block as input. The calculation formula is as follows:


(8) $$\begin{array}{l} \begin{array}{c} x_{i}=x_{i-1}+ReLU\left(x_{i-1},w_{i-1}\right) \end{array} \end{array}$$

where *x*_*i*_ is the output of the current residual block, *x*_*i*−1_ is the output of the last residual block, ReLU is the activation function, which can effectively improve the accuracy of the model.

### 3.3. Loss function

The aim of our training is to recover clear images with low-level and high-level features from fogged images. In order to obtain high quality images, we use a combined loss function for optimization during the training process. Therefore, given a training dataset ${\{R_{T}^{n},G^{n}\}}_{n}^{N}$ for T-stage, we solve


(9) $$\begin{array}{l} \begin{array}{c} L=\overset{t}{\underset{T=1}{\sum }}\left\{\alpha L_{C}\left(R_{T}^{n},G^{n}\right)+\beta L_{S}\left(R_{T}^{n},G^{n}\right)\right\}, \end{array} \end{array}$$

where $R_{T}^{n}$ is the outputs of stage T, and *G*^*n*^ represents the ground-truth images. The loss coefficients of α and β are set to 0.2 and 4. And *L*_*C*_ is the Charbonnier loss (Charbonnier et al., 1994), used to calculate the pixel loss between the predicted image and the ground truth. In addition, $L_{S}\left(R_{T}^{n},G^{n}\right)$ is the structural similarity loss (Wang et al., 2004), which is used to evaluate the structural similarity of the content of the two images. To avoid images suffering from distortion and low peak signal-to-noise ratio (PSNR), Ren et al. (2019) uses negative SSIM loss in an image recovery task and demonstrates the effectiveness of this loss on PSNR, SSIM and visual.

## 4. Experimental results

In this section, we first present the training details and evaluation metrics. Then, our method is compared qualitatively and quantitatively with advanced methods on multiple datasets. Finally, we conduct ablation experiments.

### 4.1. Experimental setup

The RESIDE dataset (Li et al., 2018) is a large-scale benchmark including synthetic images and real-world blurred images. The RESIDE is composed of five sub-data sets: Indoor Training Set (ITS), Outdoor Training Set (OTS), Synthetic Objective Testing Set (SOTS), Real-world Task-driven Testing Set (RTTS) and Hybrid Subjective Testing Set (HSTS) constitute. We selected 20,000 pairs and 500 pairs from SOTS as outdoor scene training set and outdoor scene test set respectively, and 2,000 pairs of real blurred images from RTTS for testing. In addition to the RESIDE dataset, we also conducted experiments on another publicly available dataset. O-HAZE (Ancuti et al., 2018) is an outdoor scene dataset proposed by NTIRE2018 Image Dehazing Challenge, including 45 pairs of real foggy images and corresponding fog-free images. These fogged images are taken by professional haze instruments, which can well record the same visual content under fog-free and fogged conditions. We choose 35 pairs as the training set, 5 pairs as the validation set, and 5 pairs as the test set.

Our network was trained on an Ubuntu environment, using the ADAM (Kingma and Ba, 2014) optimizer and on an NVIDIA RTX2080ti GPUs. The training was performed using the Pytorch framework. The initial learning rate was set to 3 × 10^−5^ and gradually decreases to 1 × 10^−6^. The network was trained for 50 epochs, and the input image size was 512 × 512 × 3.

In order to evaluate the image quality of single image defogging and compare it with other methods. We used the two most commonly used evaluation metrics in defogging methods: Peak Signal to Noise Ratio (PSNR) and structural similarity (SSIM). PSNR is a pixel-level image quality evaluation method used to measure the difference of gray values between two images. The higher the PSNR value,the lower the distortion between the evaluated image and the ground-truth image, and the better the quality; on the contrary, the poorer the quality. SSIM is a measure of covariance to determine the degree of structural similarity between images according to the degree of correlation between image pixels. The higher SSIM value, the more structure or color information the image retains, and the better the effect of the resulting image. What's more, we use the scikit-image library of python to calculation them. In addition, since there is no ground-truth image in real-world datasets, we use Fog Aware Density Evaluator (FADE) (Choi et al., 2015) to evaluate the haze density of the restored image. We also adopted the non-reference blind image quality evaluation indicators, NIQE (Mittal et al., 2012). NIQE is used to normalize the image contrast into blocks, and determine the image quality by calculating the average value of the local contrast of each block.

### 4.2. Image dehazing results

We evaluated the defogging results objectively and subjectively on different datasets, and compared the proposed defogging method with seven state-of-the-art methods, namely, MSCNN, AOD-Net, GCANet (Chen et al., 2019), MSBDN, FFA-Net, TDN (Chen et al., 2020), PMHLD (Liu et al., 2020a), DCNet (Bhola et al., 2021), and SSDN (Huang et al., 2021).

#### 4.2.1. Subjective evaluation

**Figure 5:**
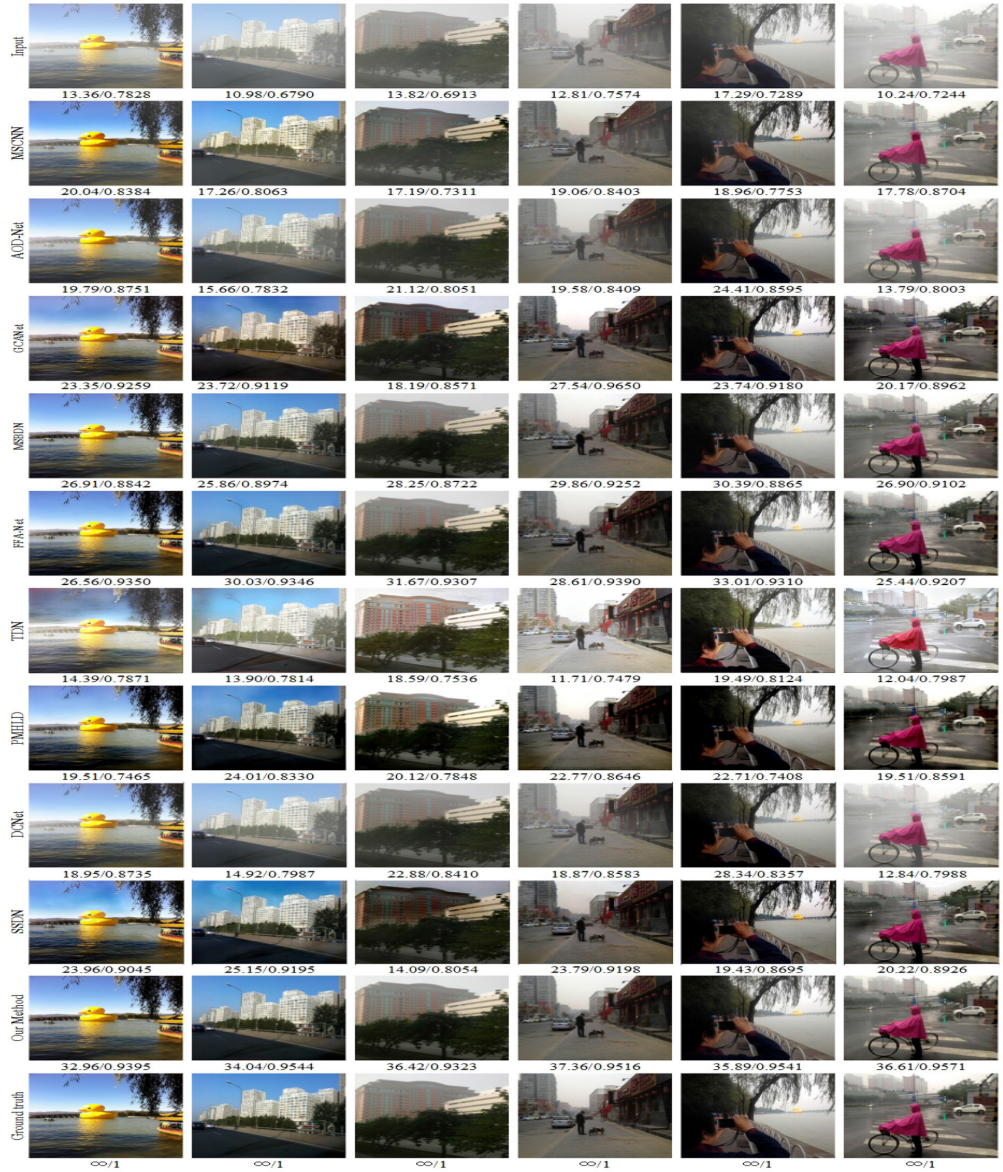
Visual results on the SOTS dataset. Best viewed on a high-resolution display.

**Figure 6:**
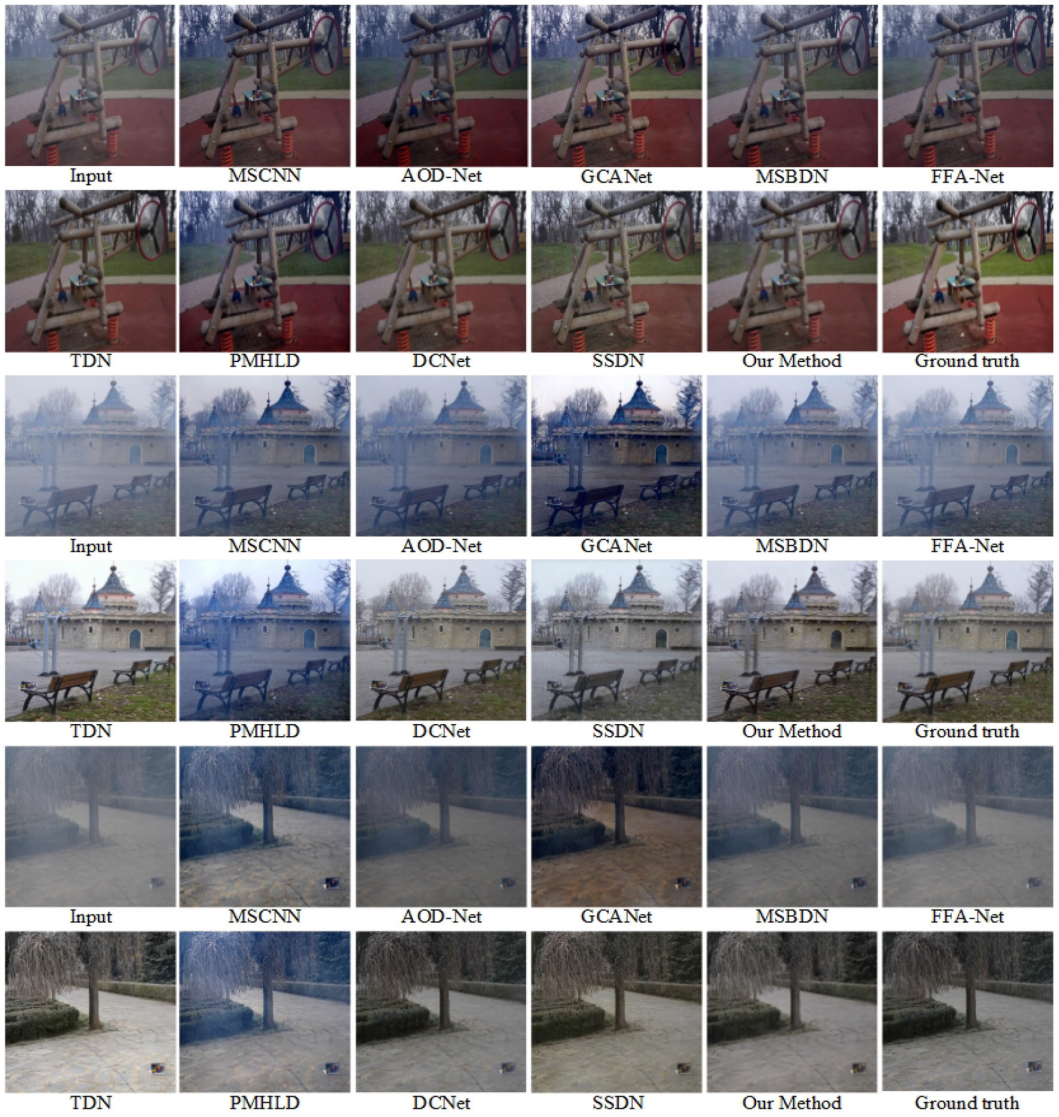
Visual results on the O-HAZE dataset. Best viewed on a high-resolution display.

**Figure 7:**
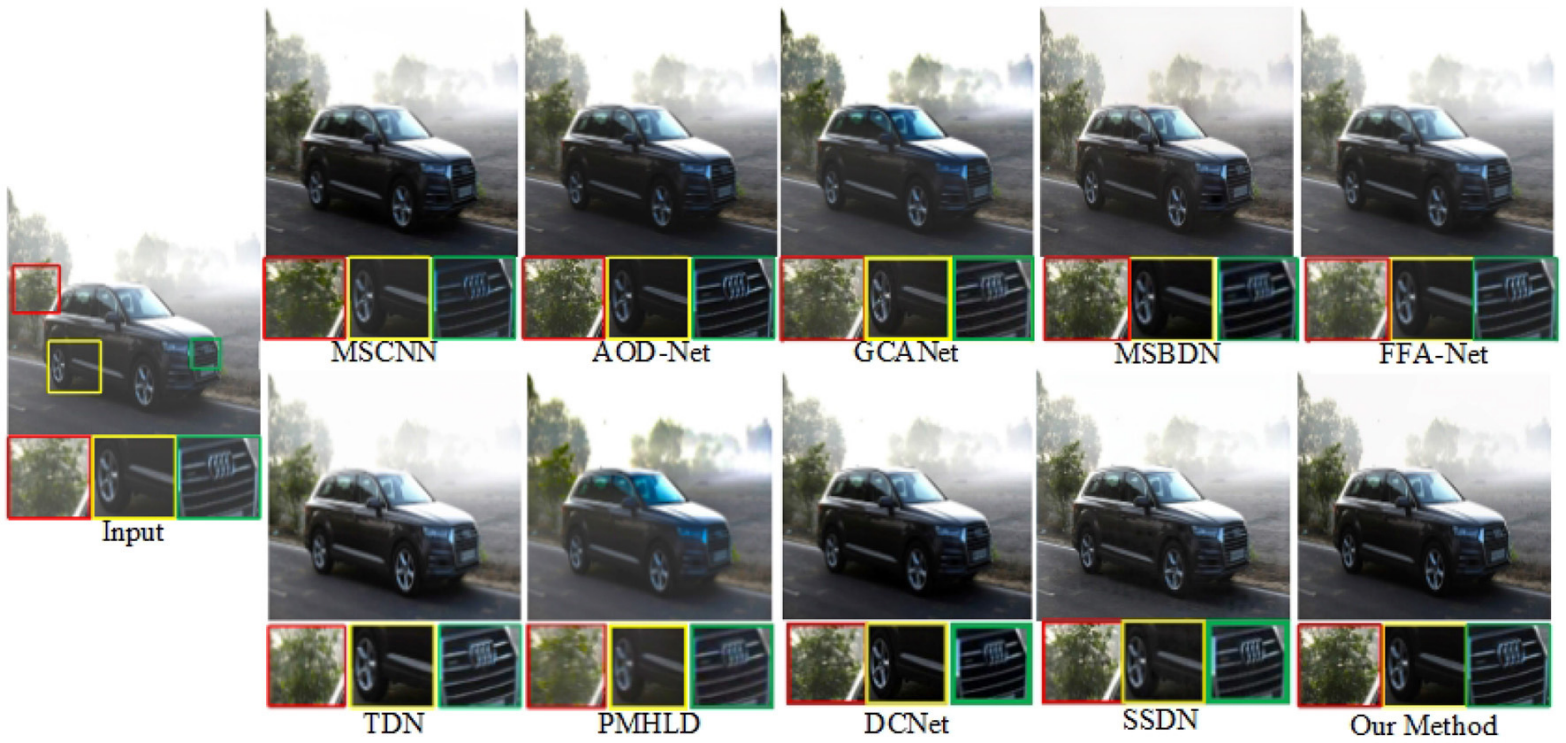
Visual quality comparison on real mist images.

**Figure 8:**
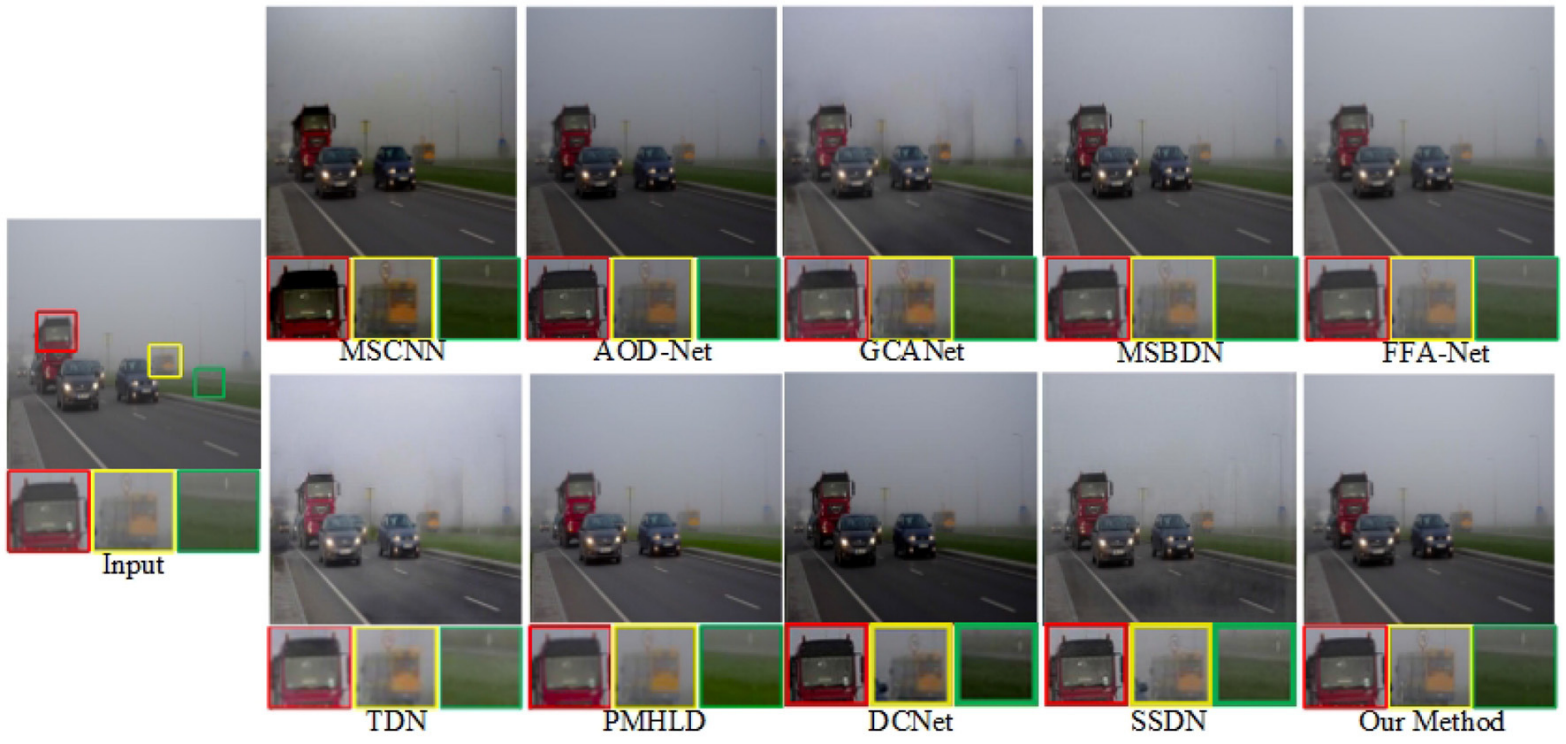
Visual quality comparison on real dense fog images.

We selected outdoor synthetic and real fogged images from the RESIDE dataset for testing, and combined our method with seven advanced methods. In addition, to verify the effectiveness of our network, we also selected real fog images from the O-HAZE dataset for testing, and selected three of them for comparison and presentation. The original fogged images, ground truth and the defogging results using 8 methods are shown in Figures 5–**8**.

In Figure 5, the top row shows the input fog image. It can be seen that MSCNN, AOD-Net and DCNet are not ideal in a slightly complex environment, and the restored colors are not bright enough. The GCA, TDN and SSDN methods have the problems of color difference, color spot and color oversaturation. MSBDN, FFA-Net, PMHLD and our methods are relatively close to the real ground images, but MSBDN and FFA-Net are not satisfactory in restoring remote scenes, while PMHLD produces color differences in the sky of column 1 and column 2. In contrast, our method performs better in color and detail in complex environments. For example, our method removes the haze around people in the fourth and fifth columns more thoroughly.

Figure 6 shows the demisting effects of different methods in the O-HAZE dataset. In the first two layers, the fog removal effect under the mist is displayed. MSCNN, AOD-Net, MSBDN, and FFA-Net not only did not remove the influence of haze, but also deepened the blurriness of the scene and made the overall color darker. Although GCANet and PMHLD reduce the fogging effect, the color of the image itself is affected, and the overall brightness of the output image is low. TDN, SSDN and our method generate more visible results with more significant demisting effect and clearer texture details.

Figures 7, 8 show the demisting effect of real scenes at different shooting distances. In these two images, the overall brightness of the images restored by MSCNN, GCA-Net, and DCNet is low, such as a large area of dark areas in the sky. The overall color of TDN, MSBDN, and FFA Net is not bright enough, and the distant scenes are not well recovered. SSDN and our method restore relatively complete details, but in the first scene, SSDN is blurred in the vegetation (red box area), and our details processing is more prominent. Compared with these advanced methods, PMHLD and our methods have more realistic details and better visibility in the restored images.

In summary, our method is visually outstanding in both synthetic and real scenes, and the recovered images are more thoroughly defogged and have clearer details such as color textures.

#### 4.2.2. Objective evaluation

In the previous section we evaluated the images after defogging through visual effects.In this section, we provide an objective analysis of several methods using two different quality evaluation metrics, PSNR and SSIM. We count the data metrics averaged over the RESIDE dataset and the O-HAZE dataset for each method and visualize them. In addition, we also show the values of SSIM and PSNR metrics for several images in Figure 5. It can be found that the PSNR values of our method are much higher than the other methods, which indicates that the less distortion and better quality between the images processed by our method and the ground-truth images. As can be observed in Table 1: our method outperforms all SOTA methods with SSIM and PSNR of 0.9438 and 33.2523 dB on the RESIDE dataset. It is intuitively seen in Figure 1 that our method significantly outperforms other methods in two metrics. In addition, the O-HAZE dataset outperforms the other methods with 0.8758dB and 24.1986dB. Compared with the RESIDE dataset, the haze in this dataset is more dense, the image quality degrades more seriously, and the defogging is more difficult, which further confirms the effectiveness of our method in a dense fog environment.

**Table 1:** Quantitatively compare the dehazing results with SOTA methods on the RESIDE and O-HAZE datasets.

**Method**	**SOTS**		**O-HAZE**	

	**SSIM**	**PSNR**	**SSIM**	**PSNR**
MSCNN	0.8436	19.49	0.7359	18.93
AOD-Net	0.8747	22.31	0.6724	18.19
GCANet	0.9151	22.89	0.6633	15.77
MSBDN	0.9068	28.64	0.6378	18.46
FFA-Net	0.9422	31.31	0.6792	18.07
TDN	0.7857	17.38	0.7286	19.41
PMHLD	0.8276	23.81	0.4839	14.40
DCNet	0.8343	19.47	0.7028	20.74
SSDN	0.8852	21.11	0.7789	25.71
**Ours**	0.9439	33.25	0.8758	24.19

Best and second best scores are red and blue. The table shows the average of the data.

Table 2 shows the objective indicators and time comparison of all methods on RTTS. NIQE, and BRISQUE evaluated the overall quality of the image. Our method obtained the best results of NIQE, indicating that the results in this paper have excellent colors and details. In terms of FADE metric, our method obtained suboptimal, while PMHLD obtained the optimal FADE value. This is inseparable from the effective haze removal of PMHLD. In terms of time, our method has only achieved the fourth place, not outstanding in efficiency.

**Table 2:** Quantitative and efficiency comparison in RTTS dataset.

**Method**	**NIQE**	**FADE**	**Runtimes**
MSCNN	3.2499	1.1716	2.3356
AOD-Net	3.4439	1.4342	0.1904
GCANet	3.2615	1.0135	0.0821
MSBDN	3.4248	1.5211	0.0394
FFA-Net	3.4515	2.0205	0.6561
TDN	3.3356	0.9217	0.8767
PMHLD	3.2254	0.7240	0.3321
DCNet	3.4188	1.2886	0.1725
SSDN	3.3756	1.8476	0.3357
Ours	3.1752	0.7873	0.4436

Color numbers indicate the best indicator value.

### 4.3. Ablation study

**Figure 9:**
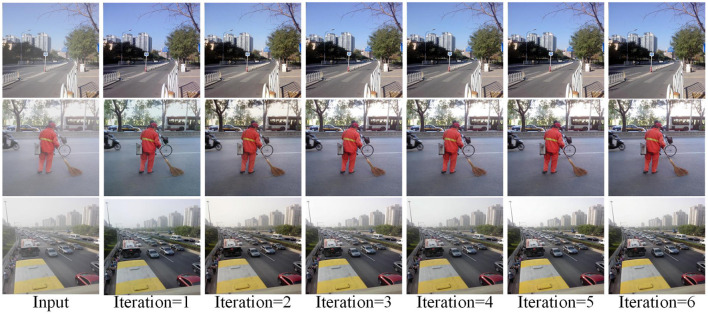
Single image defogging image obtained in different iterations.

Our approach shares the same network parameters across multiple stages through the iterative idea of using recursive computation between stages. We speculate that the defogging effect of the model will change with the increase of the number of iterations, so it is crucial to determine the optimal number of iterations.We hypothesize that the defogging effect of the model varies with the number of iterations, so it is crucial to determine the optimal number of iterations. We trained the model using iterations 1–6 under the RESIDE dataset, and Figure 9 shows the effect of image defogging under different iterations. The visual effects were similar from the 3rd to the 5th iteration, so we made an objective evaluation of these iterations. According to the comparison of PSNR and SSIM in Table 3, we found that the metrics of the third iteration and the fourth iteration were slightly lower, while the metrics of the fifth iteration and the sixth iteration were similar. By comparing the time, we choose the fifth iteration as the optimal number of iterations.

**Table 3:** Use outdoor synthetic images to test models with different iteration times, use PSNR, SSIM, and TIME for comparison.

	**SSIM**	**PSNR**	**TIME**
Iteration=3	0.9289	32.71	0.3354
Iteration=4	0.9356	33.14	0.3863
Iteration=5	0.9438	33.25	0.4436
Iteration=6	0.9438	33.28	0.4986

The value in the table is the average of all images.

## 5. Conclusion

In this paper, we propose a new transformer-based progressive residual network (PRnet). Our method recursively invokes the residual network to gradually recover clean images under ground-truth supervision. First of all, PRnet learns the features of the input images through recurrent block, while taking care of connecting the different stages to ensure that more original image features are retained during the multi-stage feature transfer of the model. We design a codec with u-net structure in combination with swin-transformer, which can ensure that sufficient contextual semantic information and spatial features are obtained during long-distance transmission. In addition, CSSI, which can ensure the synergy and connectivity of the transformer codec. Finally, the supervised fusion module can adaptively select and fuse the image features, and transfer the attention-guided features to the next stage.In addition, we demonstrate the effectiveness of the progressive network through experiments, and our model provides high-quality defogging on multiple data sets. Nonhomogeneous de-hazing is the next topic we would like to explore with our approach, as it is crucial to study complex foggy environments in real scenarios.

## Data availability statement

The original contributions presented in the study are included in the article/supplementary material, further inquiries can be directed to the corresponding author.

## Author contributions

All authors listed have made a substantial, direct, and intellectual contribution to the work and approved it for publication.

## Funding

This research was supported by the National Natural Science Foundation of China (61772319, 62002200, 62202268, and 61972235) and Shandong Natural Science Foundation of China (ZR2021MF107 and ZR2022MA076).

## Conflict of interest

Author ZY was employed by Intgrow Education Technology. The remaining authors declare that the research was conducted in the absence of any commercial or financial relationships that could be construed as a potential conflict of interest.

## Publisher's note

All claims expressed in this article are solely those of the authors and do not necessarily represent those of their affiliated organizations, or those of the publisher, the editors and the reviewers. Any product that may be evaluated in this article, or claim that may be made by its manufacturer, is not guaranteed or endorsed by the publisher.

## References

[B1] Afshar P. Heidarian S. Naderkhani F. Oikonomou A. Plataniotis K. N. Mohammadi A. (2020). COVID-caps: a capsule network-based framework for identification of COVID-19 cases from x-ray images. Pattern Recognit Lett. 138, 638–643. 10.1016/j.patrec.2020.09.010 32958971 PMC7493761

[B2] Akbari H. Yuan L. Qian R. Chuang W.-H. Chang S.-F. Cui Y.. (2021). Vatt: transformers for multimodal self-supervised learning from raw video, audio and text. arXiv preprint arXiv:2104.11178. 10.48550/arXiv.2104.11178

[B3] Ancuti C. O. Ancuti C. Timofte R. De Vleeschouwer C. (2018). “O-haze: a dehazing benchmark with real hazy and haze-free outdoor images,” in Proceedings of the IEEE Conference on Computer Vision and Pattern Recognition Workshops (Salt Lake City, UT: IEEE), 754–762.

[B4] Anvari Z. Athitsos V. (2020). Dehaze-glcgan: unpaired single image de-hazing via adversarial training. arXiv preprint arXiv:2008.06632. 10.48550/arXiv.2008.06632

[B5] Berman D. Avidan S. Avidan S. (2016). “Non-local image dehazing,” in Proceedings of the IEEE Conference on Computer Vision and Pattern Recognition (Las Vegas, NV: IEEE), 1674–1682.

[B6] Berman D. Treibitz T. Avidan S. (2018). Single image dehazing using haze-lines. IEEE Trans. Pattern Anal. Mach. Intell. 42, 720–734. 10.1109/TPAMI.2018.2882478 30475710

[B7] Bhola A. Sharma T. Verma N. K. (2021). Dcnet: dark channel network for single-image dehazing. Mach. Vis. Appl. 32, 1–11. 10.1007/s00138-021-01173-x

[B8] Cao H. Wang Y. Chen J. Jiang D. Zhang X. Tian Q.. (2021). Swin-unet: Unet-like pure transformer for medical image segmentation. arXiv preprint arXiv:2105.05537. 10.48550/arXiv.2105.05537

[B9] Carion N. Massa F. Synnaeve G. Usunier N. Kirillov A. Zagoruyko S. (2020). “End-to-end object detection with transformers,” in European Conference on Computer Vision (Glasgow: Springer), 213–229.

[B10] Charbonnier P. Blanc-Feraud L. Aubert G. Barlaud M. (1994). “Two deterministic half-quadratic regularization algorithms for computed imaging,” in Proceedings of 1st International Conference on Image Processing, Vol. 2 (Austin, TX: IEEE), 168–172.

[B11] Chen D. He M. Fan Q. Liao J. Zhang L. Hou D.. (2019). “Gated context aggregation network for image dehazing and deraining,” in 2019 IEEE Winter Conference on Applications of Computer Vision (WACV) (Waikoloa, HI: IEEE), 1375–1383.

[B12] Chen H. Wang Y. Guo T. Xu C. Deng Y. Liu Z.. (2021a). “Pre-trained image processing transformer,” in Proceedings of the IEEE/CVF Conference on Computer Vision and Pattern Recognition (Nashville, TN: IEEE), 12299–12310.

[B13] Chen J. Lu Y. Yu Q. Luo X. Adeli E. Wang Y.. (2021b). Transunet: transformers make strong encoders for medical image segmentation. arXiv preprint arXiv:2102.04306. 10.48550/arXiv.2102.04306

[B14] Chen W.-T. Fang H.-Y. Ding J.-J. Kuo S.-Y. (2020). Pmhld: patch map-based hybrid learning dehazenet for single image haze removal. IEEE Trans. Image Process. 29, 6773–6788. 10.1109/TIP.2020.2993407

[B15] Choi L. K. You J. Bovik A. C. (2015). Referenceless prediction of perceptual fog density and perceptual image defogging. IEEE Trans. Image Process. 24, 3888–3901. 10.1109/TIP.2015.2456502 26186784

[B16] Dong H. Pan J. Xiang L. Hu Z. Zhang X. Wang F.. (2020). “Multi-scale boosted dehazing network with dense feature fusion,” in Proceedings of the IEEE/CVF Conference on Computer Vision and Pattern Recognition (Seattle, WA: IEEE), 2157–2167.

[B17] Dosovitskiy A. Beyer L. Kolesnikov A. Weissenborn D. Zhai X. Unterthiner T.. (2020). An image is worth 16x16 words: transformers for image recognition at scale. arXiv preprint arXiv:2010.*11929*. 10.48550/arXiv.2010.11929

[B18] El Helou M. Süsstrunk S. (2020). Blind universal bayesian image denoising with gaussian noise level learning. IEEE Trans. Image Process. 29, 4885–4897. 10.1109/TIP.2020.2976814 32149690

[B19] Gao J. Gong M. Li X. (2021). Congested crowd instance localization with dilated convolutional swin transformer. arXiv preprint arXiv:2108.00584. 10.1016/j.neucom.2022.09.113

[B20] He K. Sun J. Tang X. (2010). Single image haze removal using dark channel prior. IEEE Trans. Pattern Anal. Mach. Intell. 33, 2341–2353. 10.1109/TPAMI.2010.168 20820075

[B21] Huang L. Tan J. Liu J. Yuan J. (2020a). “Hand-transformer: non-autoregressive structured modeling for 3D hand pose estimation,” in European Conference on Computer Vision (Glasgow: Springer), 17–33.

[B22] Huang L. Tan J. Meng J. Liu J. Yuan J. (2020b). “Hot-net: non-autoregressive transformer for 3D hand-object pose estimation,” in Proceedings of the 28th ACM International Conference on Multimedia (Seattle), 3136–3145.

[B23] Huang P. Zhao L. Jiang R. Wang T. Zhang X. (2021). Self-filtering image dehazing with self-supporting module. Neurocomputing 432, 57–69. 10.1016/j.neucom.2020.11.039

[B24] Huang Z. Li J. Hua Z. Fan L. (2022). Underwater image enhancement via adaptive group attention-based multiscale cascade transformer. IEEE Trans. Instrum. Meas. 71, 1–18. 10.1109/TIM.2022.3189630

[B25] Kar A. Dhara S. K. Sen D. Biswas P. K. (2020). Transmission map and atmospheric light guided iterative updater network for single image dehazing. arXiv preprint arXiv:2008.01701. 10.48550/arXiv.2008.01701

[B26] Kim J. Lee J. K. Lee K. M. (2016). “Deeply-recursive convolutional network for image super-resolution,” in Proceedings of the IEEE Conference on Computer Vision and Pattern Recognition (Las Vegas, NV: IEEE), 1637–1645.

[B27] Kingma D. P. Ba J. (2014). Adam: a method for stochastic optimization. arXiv preprint arXiv:1412.6980. 10.48550/arXiv.1412.6980

[B28] Li B. Ren W. Fu D. Tao D. Feng D. Zeng W.. (2018). Benchmarking single-image dehazing and beyond. IEEE Trans. Image Process. 28, 492–505. 10.1109/TIP.2018.2867951 30176593

[B29] Li J. Feng X. Hua Z. (2021). Low-light image enhancement via progressive-recursive network. IEEE Trans. Circ. Syst. Video Technol. 31, 4227–4240. 10.1109/TCSVT.2021.304994030998467

[B30] Liang J. Cao J. Sun G. Zhang K. Van Gool L. Timofte R. (2021). Swinir: image restoration using swin transformer. arXiv preprint arXiv:2108.10257. 10.1109/ICCVW54120.2021.00210

[B31] Lin A. Chen B. Xu J. Zhang Z. Lu G. (2021a). Ds-transunet: dual swin transformer u-net for medical image segmentation. arXiv preprint arXiv:2106.06716. 10.1109/TIM.2022.3178991

[B32] Lin K. Wang L. Liu Z. (2021b). “End-to-end human pose and mesh reconstruction with transformers,” in Proceedings of the IEEE/CVF Conference on Computer Vision and Pattern Recognition (Nashville, TN: IEEE), 1954–1963.

[B33] Liu J. Wu H. Xie Y. Qu Y. Ma L. (2020a). “Trident dehazing network,” in Proceedings of the IEEE/CVF Conference on Computer Vision and Pattern Recognition Workshops (Seattle, WA: IEEE), 430–431.

[B34] Liu Q. Qin Y. Xie Z. Cao Z. Jia L. (2020b). An efficient residual-based method for railway image dehazing. Sensors 20, 6204. 10.3390/s20216204 33143354 PMC7662381

[B35] Liu X. Ma Y. Shi Z. Chen J. (2019). “Griddehazenet: Attention-based multi-scale network for image dehazing,” in Proceedings of the IEEE/CVF International Conference on Computer Vision (Seoul: IEEE), 7314–7323.

[B36] Liu Z. Lin Y. Cao Y. Hu H. Wei Y. Zhang Z.. (2021). Swin transformer: Hierarchical vision transformer using shifted windows. arXiv preprint arXiv:2103.14030. 10.1109/ICCV48922.2021.00986

[B37] Middleton W. E. K. (2019). Vision Through the Atmosphere. Toronto: University of Toronto Press.

[B38] Mittal A. Soundararajan R. Bovik A. C. (2012). Making a “completely blind” image quality analyzer. IEEE Signal Process Lett. 20, 209–212. 10.1109/LSP.2012.2227726

[B39] Parmar N. Vaswani A. Uszkoreit J. Kaiser L. Shazeer N. Ku A.. (2018). “Image transformer,” in International Conference on Machine Learning (Macao), 4055–4064.

[B40] Qin X. Wang Z. Bai Y. Xie X. Jia H. (2020). “Ffa-net: Feature fusion attention network for single image dehazing,” in Proceedings of the AAAI Conference on Artificial Intelligence, Vol. 34 (New York, NY), 11908–11915.

[B41] Ren D. Zuo W. Hu Q. Zhu P. Meng D. (2019). “Progressive image deraining networks: a better and simpler baseline,” in Proceedings of the IEEE/CVF Conference on Computer Vision and Pattern Recognition (Long Beach, CA: IEEE), 3937–3946.

[B42] Ren W. Pan J. Zhang H. Cao X. Yang M.-H. (2020). Single image dehazing via multi-scale convolutional neural networks with holistic edges. Int. J. Comput. Vis. 128, 240–259. 10.1007/s11263-019-01235-8

[B43] Vaswani A. Shazeer N. Parmar N. Uszkoreit J. Jones L. Gomez A. N.. (2017). “Attention is all you need,” in Advances in Neural Information Processing Systems (Long Beach, CA), 5998–6008.

[B44] Wang C. Wu Y. Su Z. Chen J. (2020). “Joint self-attention and scale-aggregation for self-calibrated deraining network,” in Proceedings of the 28th ACM International Conference on Multimedia (Seattle), 2517–2525.

[B45] Wang N. Cui Z. Su Y. Li A. (2021). Rgnam: recurrent grid network with an attention mechanism for single-image dehazing. J. Electron. Imaging 30, 033026. 10.1117/1.JEI.30.3.033026

[B46] Wang Z. Bovik A. C. Sheikh H. R. Simoncelli E. P. (2004). Image quality assessment: from error visibility to structural similarity. IEEE Trans. Image Process. 13, 600–612. 10.1109/TIP.2003.819861 15376593

[B47] Wu B. Xu C. Dai X. Wan A. Zhang P. Yan Z.. (2020). Visual transformers: token-based image representation and processing for computer vision. arXiv preprint arXiv:2006.03677. 10.48550/arXiv.2006.03677

[B48] Xie Z. Lin Y. Yao Z. Zhang Z. Dai Q. Cao Y.. (2021). Self-supervised learning with swin transformers. arXiv preprint arXiv:2105.04553. 10.48550/arXiv.2105.04553

[B49] Yamak P. T. Yujian L. Gadosey P. K. (2019). “A comparison between arima, lstm, and gru for time series forecasting,” in Proceedings of the 2019 2nd International Conference on Algorithms, Computing and Artificial Intelligence (Sanya), 49–55.

[B50] Yan J. Li C. Zheng Y. Xu S. Yan X. (2020). Mmp-net: a multi-scale feature multiple parallel fusion network for single image haze removal. IEEE Access 8, 25431–25441. 10.1109/ACCESS.2020.2971092

[B51] Yeh C.-H. Huang C.-H. Kang L.-W. (2019). Multi-scale deep residual learning-based single image haze removal via image decomposition. IEEE Trans. Image Process. 29, 3153–3167. 10.1109/TIP.2019.2957929 31831420

[B52] Yue X. Sun S. Kuang Z. Wei M. Torr P. Zhang W.. (2021). Vision transformer with progressive sampling. arXiv preprint arXiv:2108.01684. 10.1109/ICCV48922.2021.00044

[B53] Zamir S. W. Arora A. Khan S. Hayat M. Khan F. S. Yang M.-H.. (2021). “Multi-stage progressive image restoration,” in Proceedings of the IEEE/CVF Conference on Computer Vision and Pattern Recognition (Nashville, TN: IEEE), 14821–14831.

[B54] Zhang T. Li J. Fan H. (2022). Progressive edge-sensing dynamic scene deblurring. Comput. Vis. Media 8, 495–508. 10.1007/s41095-021-0246-4

[B55] Zheng S. Lu J. Zhao H. Zhu X. Luo Z. Wang Y.. (2021). “Rethinking semantic segmentation from a sequence-to-sequence perspective with transformers,” in Proceedings of the IEEE/CVF Conference on Computer Vision and Pattern Recognition (Nashville, TN: IEEE), 6881–6890.

[B56] Zhu Q. Mai J. Shao L. (2015). A fast single image haze removal algorithm using color attenuation prior. IEEE Trans. Image Process. 24, 3522–3533. 10.1109/TIP.2015.2446191 26099141

[B57] Zhu X. Su W. Lu L. Li B. Wang X. Dai J. (2020). Deformable detr: deformable transformers for end-to-end object detection. arXiv preprint arXiv:2010.04159. 10.48550/arXiv.2010.04159

